# Supplementary material for: Effect of Xylo-Oligosaccharides Supplementation by Drinking Water on the Bone Properties and Related Calcium Transporters in Growing Mice
Source: Nutrients. 2020 Nov 19;12(11):3542. doi: 10.3390/nu12113542 (PMC7699350; doi:10.3390/nu12113542)
Supplement: Supplementary file 1 [file nutrients-12-03542-s001.pdf]

nutrients

MDPI

Supplementary Material

Table S1. Bone mineral content (BMC) of 38, 48 and 58 days mice fed different levels of XOS (Mean values and standard errors; *n* = 8 in each group).

Variables	Age(days)	0%XOS		1%XOS		2%XOS		4%XOS	
		Mean	SE	Mean	SE	Mean	SE	Mean	SE
BMC (*g*)	38	0.030	0.001	0.029	0.001	0.030	0.002	0.029	0.001
	48	0.036	0.001	0.035	0.002	0.035	0.001	0.038	0.001
	58	0.038	0.002	0.038	0.001	0.038	0.001	0.040	0.002

SE, standard error; BMC, bone mineral content [a, b] Mean values with unlike superscript letters within the same row are significantly different ($p < 0.05$; Tukey *post hoc* test). no letter = no significance.
